# Different temporal windows for CB1 receptor involvement in contextual fear memory destabilisation in the amygdala and hippocampus

**DOI:** 10.1371/journal.pone.0205781

**Published:** 2019-01-15

**Authors:** Jonathan L. C. Lee, Felippe E. Amorim, Lindsey F. Cassini, Olavo B. Amaral

**Affiliations:** 1 University of Birmingham, School of Psychology, Edgbaston, Birmingham, United Kingdom; 2 Institute of Medical Biochemistry Leopoldo de Meis, Federal University of Rio de Janeiro, Rio de Janeiro, Brazil; Universita degli Studi di Roma La Sapienza, ITALY

## Abstract

Reconsolidation is a process in which re-exposure to a reminder causes a previously acquired memory to undergo a process of destabilisation followed by subsequent restabilisation. Different molecular mechanisms have been postulated for destabilisation in the amygdala and hippocampus, including CB1 receptor activation, protein degradation and AMPA receptor exchange; however, most of the amygdala studies have used pre-reexposure interventions, while those in the hippocampus have usually performed them after reexposure. To test whether the temporal window for destabilisation is similar across both structures, we trained Lister Hooded rats in a contextual fear conditioning task, and 1 day later performed memory reexposure followed by injection of either the NMDA antagonist MK-801 (0.1 mg/kg) or saline in order to block reconsolidation. In parallel, we also performed local injections of either the CB1 antagonist SR141716A or its vehicle in the hippocampus or in the amygdala, either immediately before or immediately after reactivation. Infusion of SR141716A in the hippocampus prevented the reconsolidation-blocking effect of MK-801 when performed after reexposure, but not before it. In the amygdala, meanwhile, pre-reexposure infusions of SR141716A impaired reconsolidation blockade by MK-801, although the time-dependency of this effect was not as clear as in the hippocampus. Our results suggest the temporal windows for CB1-receptor-mediated memory destabilisation during reconsolidation vary between brain structures. Whether this reflects different time windows for engagement of these structures or different roles played by CB1 receptors in destabilisation across structures remains an open question for future studies.

## Introduction

Memory reconsolidation is a core process in the maintenance and updating of long-term memories [1]. Reexposure to reminders reactivates previously learned memories, which may lead not only to their behavioural expression but also to reconsolidation [2]. As reconsolidation depends upon neurochemical and cellular mechanisms of synaptic plasticity, such as NMDA receptor activation [3] and protein synthesis [4], pharmacological treatment around the time of memory reactivation can disrupt reconsolidation and result in subsequent amnesia.

Importantly, memory reactivation does not necessarily trigger reconsolidation [5]. Instead there is a necessity for synaptic destabilisation, which has been shown to be dissociable from behavioural expression of the memory [6–8]. Activation of GluN2B-containing NMDA receptors in the basolateral amygdala (BLA) was required for the destabilisation of auditory cued fear memories, whereas antagonism of AMPA or GluN2A-containing NMDA receptors selectively disrupted expression of the cued fear [6, 7]. Beyond NMDA receptor activation, other proposed neurochemical mechanisms of memory destabilisation in the BLA include dopamine receptors [9], cellular processes such as AMPA receptor subunit exchange [10], synaptic protein degradation [11, 12] and autophagy [12], and enzymes such as calcineurin [13], PP1 [14] and CaMKII [15] (Table 1).

**Table 1:** Involvement of different molecular mechanisms in memory destabilisation before and after its reactivation in the amygdala.

Timing	Target	Reference	Task	Species	Drug	Infusion Time	Reconsolidation Inhibitor		Outcome		
Pre-reexposure	NMDA	[6]	AFC	Rat	AP5 / Ifenprodil	10 min		Anisomycin		Blocks destabilisation	
		[7]	AFC	Rat	Ifenprodil	Immediately		Anisomycin		Blocks destabilisation	
		[22]	CFC	Mouse	Ifenprodil	5 min		Anisomycin		Blocks destabilisation	
		[14]	CPP	Mouse	MK-801 / Ifenprodil	30 min		Anisomycin		Blocks destabilisation	
		[14]	CPP	Mouse	NVP-AAM077	30 min		Anisomycin		No effect	
	AMPA	[23]	CTA	Rat	NBQX	20 min		Anisomycin		Impairs retrieval	
		[7]	AFC	Rat	LY293558	Immediately		Anisomycin		No effect	
	GluA2 endocytosis	[10]	AFC	Rat	Tat-GluA2_3Y_	60 min		Anisomycin / NASPM		Blocks destabilisation	
		[12]	AFC	Rat	Tat-GluA2_3Y_	15 min		Anisomycin + tBC		Blocks destabilisation	
	Calcineurin	[13]	IA	Mouse	FK506	5 min		Anisomycin		Blocks destabilisation and memory enhancement	
		[14]	CPP	Mouse	CyA / FK506	30 min		Anisomycin		Blocks destabilisation	
	Dopamine receptors	[9]	AC	Rat	SCH23390 / Raclopride	Immediately		Anisomycin		Blocks destabilisation	
		[9]	AC	Rat	α-flupenthixol	Immediately		Anisomycin		No effect
	PP1	[14]	CPP	Mouse	Calyculin A / Okadaic acid	30 min		Anisomycin		Blocks destabilisation
	Autophagy	[12]	AFC	Rat	Spautin-1	15 min		Anisomycin		Partially blocks destabilisation
		[12]	AFC	Rat	tBC	15 min		Anisomycin		Enhances destabilisation
Post-reexposure	NMDA	[6]	AFC	Rat	AP5 / Ifenprodil	Immediately		Anisomycin		No effect
	UPS	[11]	CFC / AFC	Rat	β-lactacystin	Immediately		Anisomycin		Blocks destabilisation
		[13]	IA	Mouse	β-lactacystin	Immediately		Anisomycin		Blocks destabilization and memory ennhancement
	CaMKII	[15]	CFC	Rat	Myr-AIP	Immediately		Anisomycin		Blocks destabilisation

Table shows the time of infusion, target molecule, reference, behavioural task, species, drugs used to block reconsolidation and destabilisation and behavioural outcome. All reconsolidation and labilization blockers were injected in the amygdala. α-flupenthixol, non-subtype selective dopamine receptor antagonist; β-lactacystin, clasto-Lactacystin-b-lactone; AC, appetitive conditioning; AFC, auditory fear conditioning; AMPA, α-amino-3-hydroxy-5-methyl-4-isoxazolepropionic acid; AP5, 2-amino-5-phosphopentanoic acid, NMDA antagonist; Calyculin A, PP1 inhibitor; CaMKII, calcium–calmodulin dependent protein kinase II; CFC, contextual fear conditioning; CPP, conditioned place preference; CTA, conditioned taste aversion; CyA, Cyclosporin A, calcineurin inhibitor; FK-506, calcineurin inhibitor; GluA2, A2 subunit of the AMPA receptor; IA, inhibitory avoidance; Ifenprodil, GluN2B-containing NMDA receptor antagonist; LY293558, AMPA receptor antagonist; MK-801, dizolcipine, NMDA antagonist; Myr-AIP, myristoylated autocamtide-2 related inhibitory peptide, CaMKII inhibitor; NASPM, 1-naphthyl acetyl spermine, Ca^++^-permeable AMPA receptor antagonist; NBQX, 2,3-dihydroxy-6-nitro-7-sulfamoyl-benzo[f]quinoxaline AMPA receptor antagonist; NMDA, N-methyl D-aspartate; NVP-AAM077, GluN2A-containing NMDA receptor antagonist; Okadaic acid, PP1 inhibitor; PP1, protein phosphatase 1; Raclopride, D2 receptor antagonist; SCH23390, D1 receptor antagonist; Spautin-1, inhibitor of ubiquitin-specific peptidases 10 and 13; Tat-GluA2_3Y_, interference peptide disrupting GluA2 endocytosis; tBC, retro-inverso Tat-beclin 1 peptide D-amino acid sequence, autophagy inducer; UPS, ubiquitin-proteasome system.

The requirement of NR2B activation, AMPA receptor endocytosis and proteasome-mediated protein degradation recapitulate findings also observed in hippocampal contextual fear memory destabilisation [16, 17], suggesting that there are common mechanisms of memory destabilisation across neural loci. Other mechanisms proposed to mediate destabilisation in the hippocampus include activation of CB1 receptors and L-type-voltage-dependent calcium channels (LVGCCs) [18, 19] (Table 2). Given that contextual fear conditioning relies critically upon both the dorsal hippocampus (DH) and the BLA [20, 21], synaptic destabilisation is likely to occur in both loci, putatively acting upon local plasticity mechanisms that are activated during memory reactivation.

**Table 2:** Involvement of different molecular mechanisms in memory destabilisation before and after its reactivation in the hippocampus.

Timing	Target	Reference	Task	Species	Drug	Infusion Time	Reconsolidation Inhibitor	Outcome
Pre-reexposure	mGluRs	[28]	CFC	Rat	3HPG	10 min	ACEA + Sulfasalazine	No effect
	Na+ channels	[28]	CFC	Rat	Tetrodotoxin	10 min	ACEA + Sulfasalazine	Blocks destabilisation
	Dopamine receptors	[29]	OR	Rat	SCH23390	15 min	Anisomycin/α-amanitin	Blocks destabilisation
	NMDA	[30]	CFC	Rat	Ifenprodil	15 min	Distractor stimulus	Blocks destabilisation
Post-reexposure	UPS	[24]	CFC	Rat	β-lactacystin	Immediately	Anisomycin	Blocks destabilisation and memory enhancement
		[16]	CFC	Mouse	β-lactacystin	Immediately	Anisomycin	Blocks destabilisation
		[25]	MWM	Rat	β-lactacystin	Immediately	Anisomycin	Blocks destabilisation
		[26]	CFC	Mouse	MG132	Immediately	Sulfasalazine	Blocks destabilisation
	CB1	[19]	CFC	Mouse	SR14716A	Immediately	Anisomycin	Blocks destabilisation
		[18]	MWM	Mouse	SR14716A	Immediately	Anisomycin	Blocks destabilisation
	LVGCCs	[19]	CFC	Mouse	Verapamil	Immediately	Anisomycin	Blocks destabilisation
		[18]	MWM	Mouse	Verapamil	Immediately	Anisomycin	Blocks destabilisation

Table shows the time of infusion, target molecule, reference, behavioural task, species, drugs used to block reconsolidation and destabilisation and behavioural outcome. All reconsolidation and labilization blockers were injected in the hippocampus. α-amanitin, selective inhibitor of RNA polymerase II and III; 3HPG, (*S*)-3-Hydroxyphenylglycine, mGluR1 agonist; ACEA, arachidonyl-2-chloroethylamide, CB1 agonist; CREB, cAMP response element-binding protein; LVGCCs, L-type voltage-gated calcium channels; MG132, (N-[(phenylmethoxy)carbonyl]-L-leucyl-N-[(1S)-1-fromyl-3-methylbutyl]-L-leucinamide, proteasome inhibitor; MWM, Morris Water Maze; OR, object recognition; SR14716A, rimonabant, CB1 antagonist; Sulfasalazine, IKappaB kinase inhibitor; Tetrodotoxin, sodium channel blocker; Verapamil, L-type calcium channel inhibitor. Drugs and abbreviations not described here are shown in the legend to Table 1.

The temporal requirement for destabilisation mechanisms in both structures, however, has not been extensively explored. Initially, it was shown that infusion of the GluN2B receptor antagonist ifenprodil into the BLA successfully disrupted memory destabilisation only when performed pre-reactivation [6]. Pre-reactivation infusions have been the norm in exploring destabilisation mechanisms is the amygdala (Table 1), with only a handful of studies describing destabilisation-blocking effects of drugs injected post-reactivation–in this case, targeting intracellular processes such as protein degradation [11, 13] and CaMKII activation [15].

On the contrary, most observations of destabilisation-blocking effects in the hippocampus have been observed with post-training interventions, including protein degradation inhibitors [16, 24–26] but also CB1 receptor antagonists and LVGCC blockers [19] (Table 2). The involvement of neurotransmitter receptors at a later stage of the process than that observed in the amygdala suggests that the temporal dynamics of destabilisation might differ between these structures. However, this comparison is complicated by the fact that these studies differed not only in the locus of drug infusion, but also in the neurochemical target and the type of fear conditioning assessed (i.e. cued vs contextual).

To address this question, we aimed to directly compare the effects of pre- and post-reactivation pharmacological interventions in the BLA and DH on the destabilisation of contextual fear memory. As our established reconsolidation-blocking drug, MK-801 [4] is a non-competitive NMDA receptor antagonist, we chose not to target GluN2B-related mechanisms, and instead focussed on CB1 receptor involvement in destabilisation [27, 28].

## Methods

### Systematic review of the literature on destabilisation mechanisms

To review the literature summarised in Tables 1 and 2, we performed a PubMed search for (“destabilization” OR “destabilisation” OR “labilization” OR “labilisation” OR "labile" OR "stability") AND (“reconsolidation” OR "retrieval" OR "recall" OR "reactivation") AND ("hippocampus" OR "amygdala" OR "hippocampal" OR "amygdalar" OR "BLA" OR "intrahippocampal" OR "intraamygdala"). Our search yielded 166 articles, which were screened to select those that studied the behavioural effects of a drug injected in the amygdala or hippocampus on reconsolidation blockade caused by another intervention performed before or after memory reactivation. This led to the inclusion of 19 studies (with an additional study added manually), for which we extracted data regarding the structure, behavioural task, species, drugs and/or interventions, injection timing and behavioural outcome (Tables 1 and 2).

### Subjects

143 male Lister Hooded rats (275–325 g at the time of surgical preparation), were housed in quads under a 12 h light/dark cycle (lights on at 0700) at 21°C with food and water provided ad libitum except during the behavioural sessions. Standard cages contained aspen chip bedding and environmental enrichment was available in the form of a Plexiglass tunnel. Experiments took place in a behavioural laboratory between 0830 and 1500. At the end of the experiment, animals were humanely killed via a rising concentration of CO2; death was confirmed by cervical dislocation. All procedures were approved by a local ethical review committee (University of Birmingham Animal Welfare and Ethical Review Body) and conducted in accordance to the United Kingdom Animals (Scientific Procedures) Act 1986, Amendment Regulations 2012 (PPL P8B15DC34).

### Surgical preparation

All rats were implanted with chronic indwelling stainless steel cannulae (Coopers Needleworks, UK) under isoflurane anaesthesia and aseptic conditions according to our established procedures [31]. 57 rats had cannulae targeting the DH [32] and the remaining 86 rats had cannulae targeting the basolateral amygdala [33]. Cannula placements were verified by Nissl-staining of sectioned drop-perfused brains. Rats were included in the data analysis if there was histological evidence (glial scars) for the injector tip being located within the DH (including CA1, DG & CA3) or BLA (including all subregions of the Lateral Amygdaloid Nucleus and Basolateral Amygdaloid Nucleus). 9 rats were excluded from the DH groups, all on the basis of histological assessment. 37 rats were excluded from the BLA groups; 15 could not be infused bilaterally, and 22 were excluded on histological basis.

### Drugs

MK-801 (Abcam, UK) was dissolved in sterile saline to a concentration of 0.1 mg/ml and was administered i.p. at a dose of 0.1 mg/kg [27]. SR141716A (Tocris, UK) was dissolved in a vehicle solution containing 3 drops (~100 μl) of Tween 80 in 2.5 mL of 7.5% dimethylsulphoxide in PBS to a concentration of 8 μg/μl. Intracranial infusions were conducted using 28G cannulae connected to an infusion pump by polyethylene tubing. 1.0 μl/side was infused into the DH and 0.5 μl/side was infused into the BLA.

### Behavioural equipment

The conditioning chambers (MedAssociates, VT) consisted of two identical illuminated boxes (25 cm × 32 cm × 25.5 cm), placed within sound-attenuating chambers. The box walls were constructed of steel, except by the ceiling and front wall, which were made of perspex. The grid floor consisted of 19 stainless steel rods (4.8 mm diameter; 1.6 mm centre-to-centre), connected to a shock generator and scrambler (MedAssociates, VT). Infrared video cameras were mounted on the ceiling of the chambers (Viewpoint Life Sciences, France) and used to record and quantify freezing behaviour automatically.

### Behavioural procedures

Rats were conditioned and tested in pairs using previously-established behavioural parameters [27]. In the conditioning session, they received 2 unsignalled footshocks (0.7 mA, 1.5-s), delivered 180 s and 211.5 s into a 273-s session. Two days later, they were returned to the conditioning chamber for a 5-min reactivation session. Rats were infused with SR141716A or vehicle into the DH or BLA either immediately before or immediately after reactivation. They were also injected i.p. with MK-801 or saline, either immediately after the reactivation session (for the pre-reactivation infusion condition) or immediately after the post-reactivation infusion. 3 hours after reactivation, they were returned to the conditioning chamber for a 2-min post-reactivation short-term memory (PR-STM) test. A further 2-min test was conducted 24 hr after reactivation (post-reactivation long-term memory; PR-LTM). All test sessions were video-recorded and automatically quantified for freezing behaviour using video tracking software (Viewpoint Life Sciences, France).

### Statistical analyses

% of time freezing during the test sessions was initially analysed with repeated measures 4-way ANOVA in JASP 0.8.5.1 (JASP Team 2016) with Timing (pre- vs post-reactivation infusion), Infusion (vehicle vs SR141716A), Injection (Saline vs MK-801), and Test (PR-STM vs PR-LTM) as factors. Planned comparisons analysed the effects of Timing, Infusion and Injection at PR-LTM, PR-STM and Reactivation individually using 3-way ANOVA. Any significant main effects or interactions within these 3-way ANOVAs were explored with analyses of simple main effects of the MK-801 injection (vs saline), with Timing and Infusion as moderators. This strategy allowed an analysis of whether MK-801 had amnestic effects at each test under each sub-condition of SR141716A (or vehicle) infusion.

Statistical power to detect an effect size of d = 1 (estimated on the basis of our previous work using MK-801 as a reconsolidation blocker [27] and compatible with average effect size estimates generally obtained in fear conditioning experiments [34] was calculated using G*Power 3.1.9.2. Estimated power for main effects was greater than 99% in 4-way ANOVA and 92% in 3-way ANOVA. Interaction effects involving abolishment of this effect would be thus powered at around 69% and 39%, respectively. Simple main effects analyses based on individual groups, meanwhile, had a lower statistical power at 35%.

## Results

### Dorsal hippocampus

**Fig 1:**
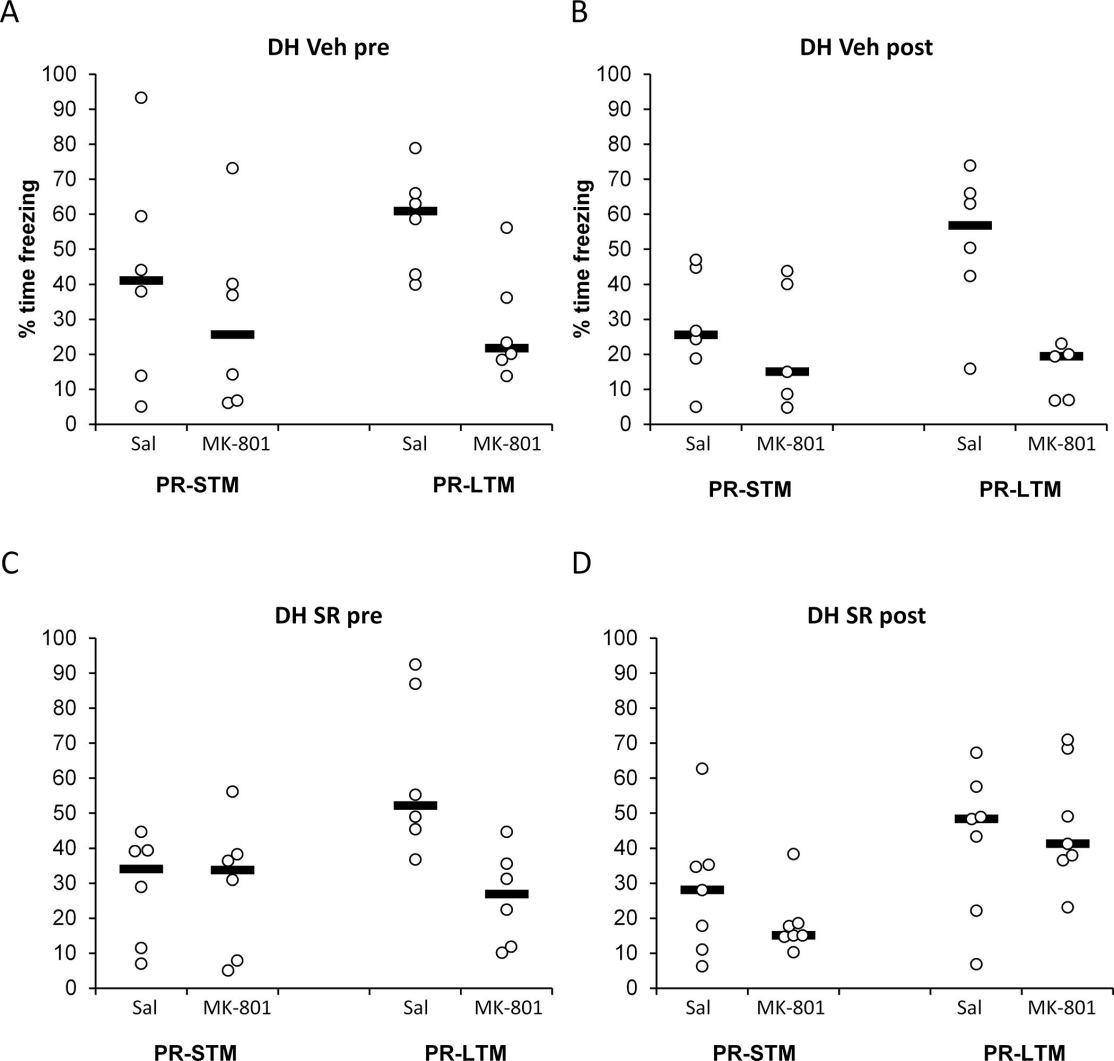
SR141716A infusion into the dorsal hippocampus blocked the amnestic effect of MK-801 when SR141716A was infused immediately after, but not before memory reactivation. MK-801 injection (0.1 mg/ml, i.p.) impaired freezing in the post-reactivation long-term memory test (PR-LTM) in rats infused with vehicle prior to (A) or immediately after (B) memory reactivation. While pre-reactivation infusion of SR141716A (8 μg/μl) did not alter the amnestic effect of MK-801 (C), post-reactivation SR141716A prevented the MK-801-induced impairment in freezing at PR-LTM (D). Statistical analyses confirmed a selective effect in the post-reactivation SR141716A condition (Timing x Infusion x Injection: F(1,41) = 5.19, p = 0.028, η^2^_p_ = 0.11). No effect of MK-801 injection or SR141716A infusion was observed in the post-reactivation short-term memory test (PR-STM). Data are presented as individual units and mean. n = 6 for all pre-reactivation groups, 6 for post-reactivation Saline, 5 for post-reactivation MK-801 and 7 for post-reactivation SR141716 + Saline and SR141716 + MK-801.

Fig 1 shows the results obtained with SR141716A infusions in the hippocampus. In the vehicle-infused groups, systemic injections of MK-801 led to a decrease in freezing in comparison to saline-injected controls in PR-LTM tests performed 24 h after reactivation, while no differences were observed in PR-STM tests. SR141716A infusion in the hippocampus abolished the MK-801 effect when performed after reactivation, but had no effect when performed before it.

Analysis of conditioned freezing at PR-STM and PR-LTM tests revealed that the amnestic effect of post-reactivation MK-801 upon contextual fear memory reconsolidation depended upon the timing of intra-dorsal hippocampus infusion of SR141716A vs vehicle (Timing x Infusion x Injection x Test: F(1,41) = 5.16, p = 0.028, η^2^_p_ = 0.11). Planned comparisons showed that, at PR-STM, there was no effect of infusion timing, SR141716A or MK-801 (Timing x Infusion x Injection: F(1,41) = 0.57, p = 0.46, η^2^_p_ = 0.01; Timing x Infusion: F(1,41) = 0.21, p = 0.65, η^2^_p_ = 0.005; Timing x Injection: F(1,41) = 0.013, p = 0.91, η^2^_p_ = 0.000; Infusion x Injection: F(1,41) = 0.16, p = 0.69, η^2^_p_ = 0.004; Timing: F(1,41) = 1.97, p = 0.17, η^2^_p_ = 0.05; Infusion: F(1,41) = 0.59, p = 0.45, η^2^_p_ = 0.01; Injection: F(1,41) = 1.33, p = 0.26, η^2^_p_ = 0.03). In contrast, at PR-LTM there was further evidence for a timing-dependent SR141716A modulation of reconsolidation disruption by MK-801 (Timing x Infusion x Injection: F(1,41) = 5.19, p = 0.028, η^2^_p_ = 0.11; Timing x Infusion: F(1,41) = 1.06, p = 0.31, η^2^_p_ = 0.03; Timing x Injection: F(1,41) = 2.67, p = 0.11, η^2^_p_ = 0.06; Infusion x Injection: F(1,41) = 3.26, p = 0.08, η^2^_p_ = 0.07; Timing: F(1,41) = 0.72, p = 0.40, η^2^_p_ = 0.02; Infusion: F(1,41) = 1.22, p = 0.28, η^2^_p_ = 0.03; Injection: F(1,41) = 22.94, p<0.001, η^2^_p_ = 0.36). Planned analyses of simple main effects of Injection, with Timing and Infusion as moderators, confirmed that there were impairments in freezing in MK-801-injected rats, compared to saline-injected controls, in both pre-reactivation infusion groups (Vehicle: p = 0.005; SR141716A: p = 0.001) and in the post-reactivation vehicle infusion group (p = 0.001), but not in the post-reactivation SR141716A group (P = 0.62). Therefore, SR141716A protected against the MK-801-induced impairment of PR-LTM only when infused immediately after the reactivation session.

The effect at PR-LTM was not obviously due to differences in initial conditioning or differences between groups at the reactivation session (Table 3). Analysis of freezing at the reactivation session revealed an overall effect of pre-reactivation infusions to reduce freezing. (i.e. all pre-reactivation groups [both saline and MK-801] vs all post-reactivation groups: F(1,41) = 4.87, p = 0.033, η^2^_p_ = 0.11). Unlike for freezing at PR-LTM, there was no Timing x Infusion x Injection interaction (F(1,41)<0.001, p = 0.98, η^2^_p_ = 0.00).

**Table 3:** Contextual freezing at the reactivation session.

			Mean	SEM
**DH**				
Pre-reactivation	Veh	Sal	26.9	13.7
		MK-801	23.2	12.1
	SR	Sal	14.6	6.0
		MK-801	13.9	5.7
Post-reactivation	Veh	Sal	52.1	12.9
		MK-801	41.6	12.6
	SR	Sal	38.6	5.7
		MK-801	31.9	6.5
**BLA**				
Pre-reactivation	Veh	Sal	32.1	4.1
		MK-801	33.5	4.4
	SR	Sal	28.3	2.6
		MK-801	37.0	10.1
Post-reactivation	Veh	Sal	36.7	3.7
		MK-801	36.2	4.0
	SR	Sal	37.1	3.7
		MK-801	28.3	3.7

### Basolateral amygdala

**Fig 2:**
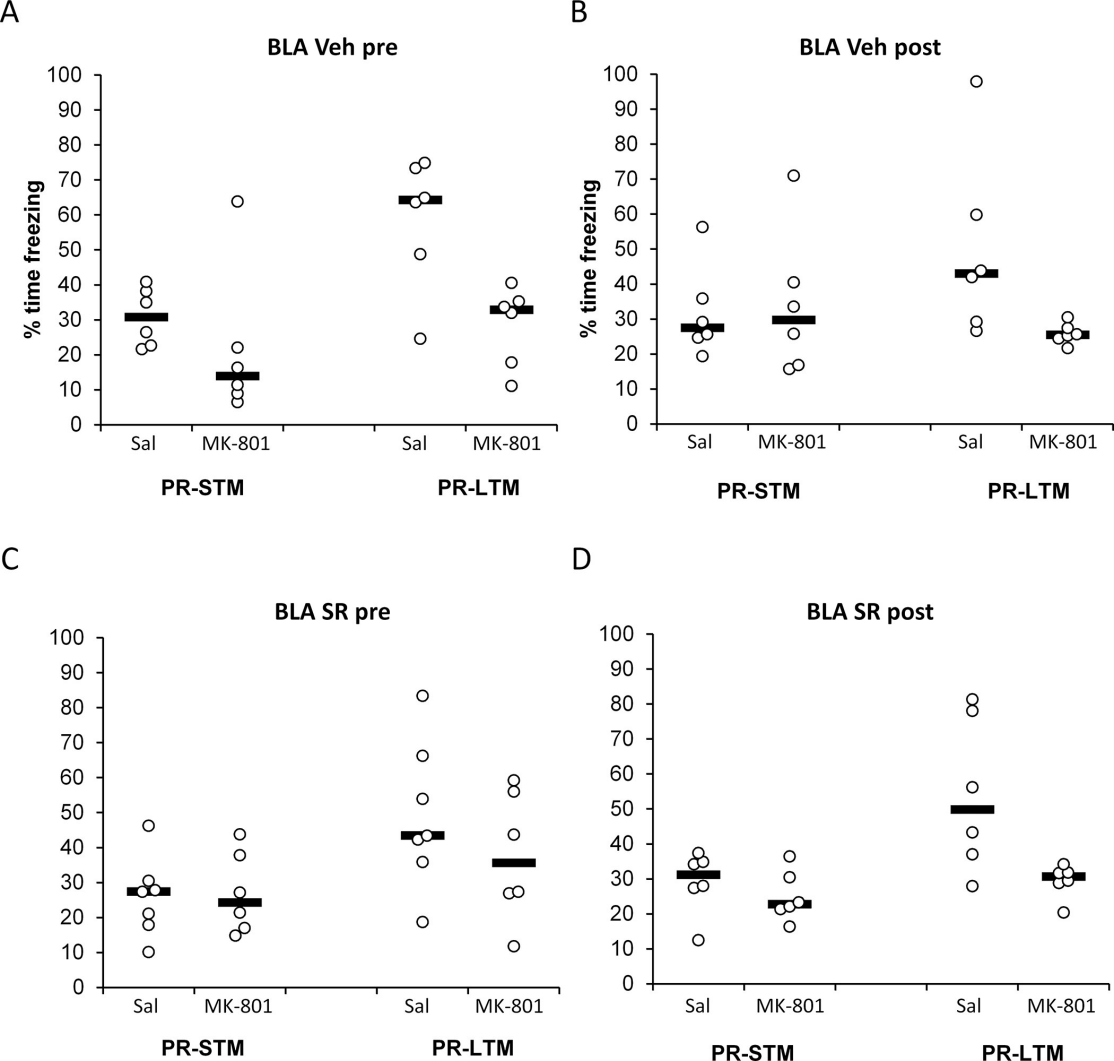
CB1 receptor antagonism in the basolateral amygdala impairs memory destabilization when performed before memory reactivation. MK-801 injection (0.1 mg/ml, i.p.) impaired memory reconsolidation in the long-term memory test (PR-LTM) in the groups infused with vehicle either before (A; simple main effect of injection, p = 0.006) or immediately after (B; p = 0.023) memory reactivation. While pre-reactivation infusion of SR141716A (8 μg/μl) seemed to prevent the amnestic effect of MK-801 (C; p = 0.25), post-reactivation infusion did not (D; p = 0.021. However, there was no significant interaction between the timing of infusion and the effect of MK-801 (Timing x Infusion x Injection: F(1,41) = 1.11, p = 0.30, η^2^_p_ = 0.026). No effect of MK-801 injection or SR141716A infusion is observed in the short-memory test at 3h (PR-STM). Data are presented as individual units and mean. n = 6 per group, except for pre-reactivation SR141716 + Saline, in which n = 7.

Fig 2 shows the results obtained with SR141716A infusions in the amygdala. Once more, systemic injections of MK-801 led to decreased freezing in PR-LTM tests in both vehicle-infused groups. Unlike in the hippocampus, however, SR141716A attenuated this effect when infused pre-reactivation, and had no effect when infused after it. Once again, no differences were observed in PR-STM tests.

Analysis of conditioned freezing at PR-STM and PR-LTM tests revealed that the amnestic effect of post-reactivation MK-801 upon contextual fear memory reconsolidation was not obviously dependent upon the timing of SR141716A infusion (Timing x Infusion x Injection x Test: F(1,41) = 0.009, p = 0.93, η^2^_p_ = 0.00; Infusion x Injection x Test: F(1,41) = 0.32, p = 0.58, η^2^_p_ = 0.008; Injection x Test: F(1,41) = 11.04, p = 0.002, η^2^_p_ = 0.21). Planned comparisons showed that at PR-STM, there was no effect of infusion timing, SR141716A or MK-801 (Timing x Infusion x Injection: F(1,41) = 1.11, p = 0.30, η^2^_p_ = 0.03; Timing x Infusion: F(1,41) = 0.60, p = 0.44, η^2^_p_ = 0.01; Timing x Injection: F(1,41) = 0.15, p = 0.70, η^2^_p_ = 0.004; Infusion x Injection: F(1,41) = 0.075, p = 0.79, η^2^_p_ = 0.002; Timing: F(1,41) = 0.88, p = 0.35, η^2^_p_ = 0.02; Infusion: F(1,41) = 0.50, p = 0.49, η^2^_p_ = 0.02; Injection: F(1,41) = 0.42, p = 0.52, η^2^_p_ = 0.02). In contrast, at PR-LTM there was evidence for an amnestic effect of MK-801 (Injection: F(1,41) = 19.77, p<0.001, η^2^_p_ = 0.33), although this was not clearly dependent upon SR141716A infusion or timing (Infusion x Injection: F(1,41) = 0.78, p = 0.38, η^2^_p_ = 0.002; Timing x Infusion x Injection: F(1,41) = 1.11, p = 0.30, η^2^_p_ = 0.026). However, planned analyses of simple main effects suggested impairments in freezing in MK-801-injected rats in both post-reactivation infusion groups (Vehicle: p = 0.021; SR141716A: p = 0.023) and the pre-reactivation vehicle infusion group (p = 0.006), but not in the pre-reactivation SR141716A group (p = 0.25). Therefore, SR141716A appeared to protect against the MK-801-induced impairment of PR-LTM when infused immediately before the reactivation session, although the dissociation between pre- and post-reactivation effects was not as clear-cut as that observed in the hippocampus.

The effect at PR-LTM was again not obviously due to differences in initial conditioning or differences between groups at the reactivation session (Table 3). Unlike for the DH, pre-reactivation infusion did not acutely affect freezing at the reactivation session (F(1,40) = 0.27, p = 0.61, η^2^_p_ = 0.007). There was also no Timing x Infusion x Injection interaction (F(1,40) = 1.20, p = 0.28, η^2^_p_ = 0.029), nor a main effect of MK-801 (F(1,40) = 0.003, p = 0.96, η^2^_p_ = 0.00, indicating that the main-effect of MK-801 on freezing at PR-LTM observed above does not seem to be due to pre-existing differences between groups.

## Discussion

Our results show that post-reactivation systemic MK-801 injection disrupted subsequent contextual fear memory expression 24h (but not 3h) after reactivation. However, this memory disruption was seemingly prevented when the CB1 receptor antagonist SR141716A was infused into the BLA or DH. Importantly, SR141716A-mediated protection against amnesia had different temporal windows of efficacy depending upon the locus of infusion. Pre-reactivation infusions were only effective when targeted into the BLA, whereas only post-reactivation infusions in the dorsal hippocampus prevented MK-801-induced amnesia.

The disruption of contextual fear memory by MK-801 likely reflects an impairment of memory reconsolidation. We and others have previously demonstrated that post-reactivation treatment with NMDA receptor antagonists disrupts reconsolidation of various types of memory [13, 35–37]. The post-reactivation timepoint of drug treatment avoids acute effects on the reactivation session itself, and the preservation of contextual fear memory expression at the 3-h post-reactivation PR-STM test, as expected for reconsolidation blockade [4], rules out non-specific chronic effects of MK-801. While we did not include an operational non-reactivation control condition, the SR141716A-induced protection against the amnestic effect of MK-801 shows that this effect is destabilisation-dependent, and thus likely dependent on reactivation. An alternative account of post-reactivation amnesia focussing on memory integration has been recently proposed [38]. While such an account might explain the amnestic effect of MK-801 alone, it is not clear how it would explain the observation that additional treatment with local SR141716A, within specific differential time windows, reverses MK-801-induced amnesia.

The prevention of MK-801-induced reconsolidation disruption by post-reactivation dorsal hippocampal SR141716A replicates a previous study in mice that used intra-hippocampal anisomycin as the amnestic agent [19]. This pattern of results, with no effect of SR141716A on its own on contextual fear memory (and no enhancement of contextual freezing that might offset the disruptive effect of MK-801) has been interpreted as an impairment of memory destabilisation [6, 19, 39, 40], and is consistent with our recent observation that pharmacological agonism of hippocampal CB1 receptors can stimulate the destabilisation of contextual fear memories [28].

While CB1 receptors in the BLA have been studied relatively extensively in fear memory and its extinction, the evidence is more limited when considering memory destabilisation/reconsolidation of contextual fear memories. The CB1 receptor antagonist AM251 had no effect on reconsolidation by itself, but prevented the enhancement of reconsolidation by CB1 receptor agonism in a fear-potentiated startle setting [41]. This was interpreted as a purely pharmacological effect (i.e. the AM251-blockade of CB1 receptors directly preventing pharmacological agonism), although it is not inconsistent with a potential effect of CB1 receptor antagonism in preventing memory destabilisation.

However, Ratano et al [42] observed that AM251, at a substantially lower dose (300 ng/side c.f. 20 μg/side), did impair post-reactivation long-term memory. This effect was shown with AM251 infusions immediately after, but not 30 min prior to, memory reactivation and appeared to be mediated by the dysregulation of GABAergic signalling in the BLA [42]. This contrasts with our observation that SR141716A had no effect alone when infused immediately before or after reactivation. It remains unclear what accounts for this discrepancy, and what are its implications for our interpretation of an intra-BLA SR141716A-mediated impairment of contextual fear memory destabilisation. A difference in memory type (cued vs contextual fear) exists, but may not be important, given that the BLA is hypothesised to have a conceptually similar role in both settings, associating the CS or contextual representation with the US [43–45]. Another difference is the CB1 antagonist employed: while AM251 and SR141716A are structurally similar, and both have “inverse cannabimimetic effects” consistent with pharmacological inverse agonism [46], there is some evidence that the two drugs may differ in their affinity for an unidentified central vanilloid VR1-like receptor [47].

Our BLA SR141716A results are not only potentially inconsistent with those of Ratano et al [42], but less clear-cut statistically than our DH SR141716A results. The conclusion that intra-BLA SR141716A disrupts contextual fear memory destabilisation only when infused prior to memory reactivation results from planned analyses of simple main effects, and should be considered as preliminary in the absence of conclusive evidence for a dependence of MK-801 effects upon either the timing of the BLA infusion or its content (SR141716A vs vehicle). Nevertheless, there is a clear difference between the BLA and DH results, with stronger evidence for intra-dorsal hippocampal SR141716A having destabilisation-impairing effects only when infused after reactivation. This temporal pattern is largely consistent with previous studies using different destabilisation-inhibitors, with pre-reactivation selectivity in the BLA [3] and post-reactivation sufficiency in the hippocampus [19, 46]. Our study adds to this picture the fact that, in the hippocampus, the effects of destabilisation blockade by CB1 receptors seem to be restricted to the post-reactivation period.

The fact that the intra-DH infusion of SR141716A immediately before reactivation did not disrupt destabilisation might indicate a delayed temporal window of CB1 receptor necessity in this structure. The short (5 min) duration of the reactivation session is such that the pharmacological action of SR141716A would likely have persisted into the immediate post-reactivation period. Indeed behavioural evidence indicates that intracranial infusions of SR141716A have acute effects lasting at least 15 min [48], which would certainly encompass both the reactivation session and at least the first part of the post-reactivation period. Alternatively, it is possible that blocking CB1 receptors before reactivation engages other mechanisms in memory destabilisation, whereas this does not occur when reactivation has already occurred. Similar accounts of ‘optional’ engaging of the hippocampus have been observed for injections prior to reactivation of remote memories [49], and although we have no clear mechanistic explanation for this hypothesis, it should nevertheless be kept in mind.

The present evidence demonstrates different temporal windows of efficacy of destabilisation blockade between the amygdala and hippocampus when the same memory task, amnestic agent and destabilisation inhibitor are used. Therefore, the differential effects are not likely to be due to pharmacodynamics or pharmacokinetics of different drugs, nor to potential differences in the engagement of destabilisation mechanisms because of discrepant memory settings and strength of conditioning. Moreover, analysis of freezing values in the reactivation session (Table 3) suggests that they are not due to pre-existing differences between groups.

This opens up an interesting question concerning whether memory destabilisation during reconsolidation represents a single, unified phenomenon, or whether distinct phenomena involving CB1 receptors in different structures lead to a similar behavioural outcome of memory weakening when reconsolidation is blocked. As destabilisation shares common molecular mechanisms across brain structures [3, 16, 50], it is possible that the different temporal profiles of CB1 involvement reflect the distinct temporal dynamics of each structure’s role in fear conditioning. In this view, destabilisation mechanisms are engaged at a later stage in the hippocampus, but are involved in plasticity processes that are similar to those in the amygdala, as suggested by the common behavioural outcome of destabilisation blockade in both structures. Such a view would allow for additional speculation about the downstream cellular mechanisms of destabilization engaged by CB1 receptor activation. Given the functional link between CB1 receptor activation and both calcineurin [51] and protein degradation at the proteasome [52], there is a likely functional pathway of memory destabilization that includes these mechanisms.

Against this view, however, is the fact that for other molecular mechanisms involved in destabilisation, this temporal dissociation does not seem as clear. Blockade of both NR2B receptors [6, 30] and D1 receptors [9, 29] has been shown to have effects on memory destabilisation when performed pre-reactivation in both the amygdala and the hippocampus. Similarly, interference with protein degradation by the proteasome is effective when performed post-training in either structure. Although one should be careful when comparing effects in different structures across different studies, as many other parameters might vary among them, this finding suggests that the temporal dissociation we have observed might be a particular feature of interventions targeted at the CB1 receptor.

A second possibility, thus, is that the mechanism through which CB1 receptor activation leads to the behavioural outcome of memory destabilisation is different in both structures. Although the pharmacological profile of destabilisation in the amygdala and in the hippocampus is generally similar, some of the molecular mechanisms involved–which include CB1 receptors, the ubiquitin-proteasome system [19, 39] and AMPA receptor endocytosis [10, 17] in both structures, L-type voltage-gated calcium channels in the hippocampus [19] and calcineurin in the amygdala [12]–are also involved in other forms of behavioural and synaptic plasticity, such as memory extinction [1], normal forgetting [53] and homeostatic synaptic downscaling [54, 55]. Thus, it is possible that the CB1 receptor might be part of a more general plasticity system that is engaged in the amygdala and the hippocampus for different purposes during memory updating.

Finally, another open question is how other functions of the CB1 receptor in the amygdala, such as the mediation of different forms of memory extinction [56, 57] and acute fear relief [58] relate to its role in memory destabilisation during contextual reexposure. It is interesting to note that CB1 receptors seem to be particularly important for within-session freezing decrease [59], a phenomenon that can temporally co-occur with memory labilization during reexposure. Although no correlation has been shown between the degree of freezing decrease during reexposure and the effect of post-reactivation injections of MK-801 [27], it is nevertheless possible that the decrease in fear during reexposure mediated by CB1 in the amygdala could play a role in setting off labilization mechanisms, which might later mediate memory updating in the hippocampus and other structures.

These and other matters, however, remain open to further studies, which might reveal whether the temporal dissociation between labilization in the hippocampus and amygdala observed with CB1 receptors occurs with other molecular targets as well. In the meantime, our results show that memory destabilisation during reconsolidation of contextual fear conditioning is a complex process that cannot be pinpointed to a single structure or time point, and that the temporal dynamics of the engagement of destabilisation mechanisms may differ between brain structures.

## Supporting information

S1 File Raw freezing data. Unit-level % time contextual freezing for the data presented in Figs 1 & 2 and Table 3.
(XLSX)
